# Permanent Interstitial Brachytherapy for Previously Irradiated Head and Neck Cancer

**DOI:** 10.7759/cureus.2517

**Published:** 2018-04-22

**Authors:** William Breen, Jacqueline Kelly, Henry S Park, Yung Son, Clarence Sasaki, Lynn Wilson, Roy Decker, Zain A Husain

**Affiliations:** 1 Department of Therapeutic Radiology, Yale University School of Medicine, New Haven, USA; 2 Otolaryngology, Yale University School of Medicine, New Haven, USA

**Keywords:** head and neck cancer, brachytherapy, re-irradiation, radiation, interstitial brachytherapy, oncology, palliative radiotherapy

## Abstract

Objective: To evaluate our institutional experience using brachytherapy for the re-irradiation of the head and neck.

Study Design/Methods: We reviewed the records of patients who received brachytherapy for head and neck cancer in a previously irradiated field between 2007 and 2016.

Results: Sixty-nine patients received brachytherapy-based re-irradiation. Forty-nine patients (71%) were treated for recurrent cancers, 15 patients (22%) had second primary cancers, and five patients (7%) were treated for persistent tumors. The median dose was 90 Gy (range 30-180). Median follow-up was 3.0 years for surviving patients and 0.6 years for all patients. Overall survival at one, three, and five years was 58%, 19%, and 12%, respectively. Local control at one, three, and five years was 55%, 38%, and 28%, respectively. A disease-free interval of less than one year was associated with significantly worse local control (p=.04). Patients who received brachytherapy for a neck disease had significantly worse locoregional control than those who received brachytherapy for mucosal disease (heart rate (HR) 2.14, 95% CI 1.00-4.56, p=.05). Patients who had an extranodal extension had significantly worse overall survival than those without an extranodal extension (HR 2.57, 95% CI 1.28-5.37, p=.008). Seventy-four percent of patients who had pain before brachytherapy (with or without surgery) had an improvement of symptoms. Acute and chronic toxicity of at least Common Terminology Criteria for Adverse Events Grade 3 was seen in 27% and 19% of the patients, respectively.

Conclusions: Brachytherapy-based re-irradiation is an effective approach for patients undergoing re-irradiation for head and neck cancer. Brachytherapy may be more effective for mucosal recurrences than neck recurrences.

## Introduction

Patients with head and neck cancer frequently have disease recurrence, with significant locoregional failure rates for patients with advanced disease treated with chemoradiation [1-2]. Second primary cancers are also common and represent the second-most common cause of death in patients with head and neck cancer [3]. In either case, the management of head and neck cancer in a previously treated area is a therapeutic challenge. Former therapies limit further management options and increase the morbidity of any potential treatment. In the case of recurrent disease, the problem is compounded by the fact that the disease in question is likely treatment refractory. Still, there is value in selecting patients for aggressive curative intent interventions, as several series have demonstrated that a minority of patients will become long-term survivors [4-7].

While salvage surgery remains the standard of care for patients with recurrent disease in previously irradiated areas, recurrence rates are substantial following surgical resection alone, with reported rates ranging from 49% to over 80% [8-9]. As demonstrated in the landmark phase III study by Janot et al., disease-free survival in these patients can be improved with chemoradiation [9]. The disease control benefits did come at a cost, however, with a two-year rate of grade three or four late toxicity of 39% in the chemoradiation arm compared to 10% in the “wait and see” arm.

Patients in that study were treated with 3-D conformal external beam radiation therapy. Given the sharper dose fall-off associated with brachytherapy, this therapy could potentially maintain similarly improved control rates while reducing long-term toxicity [10]. Unfortunately, the usage of brachytherapy has been declining in the definitive management of head and neck cancers [11]. Similarly, despite multiple studies of brachytherapy in the re-irradiation setting, it is not commonly used.

Brachytherapy has long been used at our institution as part of the treatment approach for recurrent and new primary head and neck cancers arising in previously irradiated areas, and the purpose of this study is to explore the outcomes associated with brachytherapy-based re-irradiation in a modern series of patients.

## Materials and methods

This study was approved by our Institutional Review Board and was in accordance with the Helsinki Declaration of 1975, revised in 2000. The medical records of all patients treated with brachytherapy for head and neck cancers to a previously radiated area during the era of rigorous electronic medical records were retrospectively analyzed. Sixty-nine patients met each of the following criteria and were included:

1) Pathologically confirmed cancer of the head and neck treated with brachytherapy at our institution between 2007 and 2016.

2) Brachytherapy was applied to an area previously radiated with external beam radiation therapy (EBRT) to a dose greater than 45 Gy.

3) All sites of known disease were addressed with treatment at the time of brachytherapy.

Patients were selected for brachytherapy at the discretion of individual physicians.

Brachytherapy

Brachytherapy was applied using the clinical judgment of the radiation oncologist. While preoperative imaging was used for guidance, no pre-operative dosimetry was performed. The radiation oncologist, with the help of the collaborating surgeon, sutured the brachytherapy meshes to the at-risk tissues, using either iodine-125 seeds or palladium-103 seeds. Post-operative CT scans and dosimetry were performed in the majority of cases (n=38).

Data collection

Data collected included demographics, initial imaging, initial treatment, initial pathology, human papillomavirus (HPV) status, recurrence treatment, recurrence pathology, local, regional, and distant control, and survival.

Extranodal extension (ENE) was defined as cancer extending beyond the capsule of a lymph node and was assessed with initial pathology and at recurrence. Patients in whom neck disease obliterated any remaining lymph node structure were classified as having ENE. Overall survival (OS), local control (LC), and locoregional control (LRC) were calculated from the time of brachytherapy re-irradiation. The disease-free interval before brachytherapy was calculated from the most recent local treatment to brachytherapy re-irradiation. Local control was defined as a lack of local failure. Local failure was defined as radiographic or clinical failure within two cm of the brachytherapy site. This method was chosen in order to differentiate patients who failed within the tissue directly treated by brachytherapy from those who failed outside of the brachytherapy treatment volume but within the region. Any failure elsewhere in the head and neck was considered a regional failure. Failures outside the head and neck were considered distant metastases. All sequential recurrence events were recorded (i.e., local, regional, and distant), not just initial events.

Statistical analysis

Statistical analyses were performed with the JMP software (SAS Institute Inc., Cary, North Carolina). Survival curves were created using the Kaplan-Meier method. Univariate Cox proportional hazards regression modeling was used to evaluate the association of patient and treatment factors with LC, LRC, and OS. Significance was defined as a p-value of 0.05 or less.

## Results

Sixty-nine patients were identified. Patient characteristics appear in Table 1. The median age at initial diagnosis was 58 (range 30-91). Fifty-seven (83%) patients were male. Seventy-four percent of patients were smokers. Median pack-years was 30 (range 0-90). Sixty-five patients had squamous cell carcinoma, three had acinic cell carcinoma, and one had pleomorphic sarcoma. The initial T stage was most frequently T2 (40%) and T3 (24%). The initial N stage was most frequently N0 (44%) and N2 (33%). HPV status was known in only 14 patients (seven positive). For oropharyngeal cancers, HPV status was known in only eight of 21 patients (four positive). Forty-nine patients (71%) were treated for new primaries, 15 (22%) were treated for recurrences, and five (7%) were treated for a persistent disease.

**Table 1:** Patient and Treatment Characteristics EBRT: external beam radiation therapy

Patients	n=69
Median Age	58 (30-91)
Male	n=57 (83%)
Female	n=12 (17%)
Smoking History:	n=46 (74%)
Oral Cavity Primary	n=24 (35%)
Oropharynx Primary	n=21 (30%)
Nasopharynx Primary	n=2 (3%)
Larynx/Hypopharynx	n=13 (19%)
Skin/Parotid	n=7 (10%)
Unknown Primary	n=2 (3%)
Brachytherapy for Recurrent Tumor	49 (71%)
Brachytherapy for Second Primary	15 (22%)
Brachytherapy for Persistent Tumor	5 (7%)
Initial Surgery	n=46 (67%)
Initial EBRT	n=62 (90%)
Median Initial EBRT dose (range)	63.6 Gy (45-76)
Initial Chemotherapy	n=35 (51%)
Initial Brachytherapy	n=5 (7%)
Brachytherapy for Mucosal Tumor	n=41 (59%)
Brachytherapy for neck tumor	n=24 (35%)
Brachytherapy for Mucosal and Neck Tumor	n=4 (6%)
Salvage Surgery with Intraoperative Brachytherapy	n=58 (84%)
Brachytherapy Alone	n=11 (16%)
Systemic Therapy for Recurrence	n=10 (14%)

Previous treatments

Please see Table 1 for treatment characteristics. All patients had a history of having received EBRT as part of a previous treatment course. Sixty-two patients had radiation at initial treatment; the remaining seven had radiation after recurrence. The median time between the first course of irradiation and brachytherapy was 1.8 years (range 0.12-24.8).

Brachytherapy for recurrence

Permanent interstitial brachytherapy was used for mucosal disease in 41 patients (59%), neck disease in 24 (35%), and both mucosal and neck disease in four (6%). Resection was performed at the time of brachytherapy (salvage surgery) in 58 patients (84%), while brachytherapy alone was performed in 11 patients (16%). Of the 28 patients who received neck brachytherapy, 27 (96%) had neck dissection. Of the 41 patients who received brachytherapy to mucosal disease only, 17 (41%) had neck dissection. The median brachytherapy activity was 32.4 millicuries (range 8.7-56.5). The median number of brachytherapy seeds used was 45 (range 6-95). Iodine-125 seeds were used in 43 patients (62%), while palladium-103 seeds were used in 26 patients (38%). Post-operative CT scans and dosimetry were performed in the majority of cases (n=38). The median calculated brachytherapy dose to the tumor surface was 90 Gy (range 30-180) for these patients.

Outcomes

**Figure 1:**
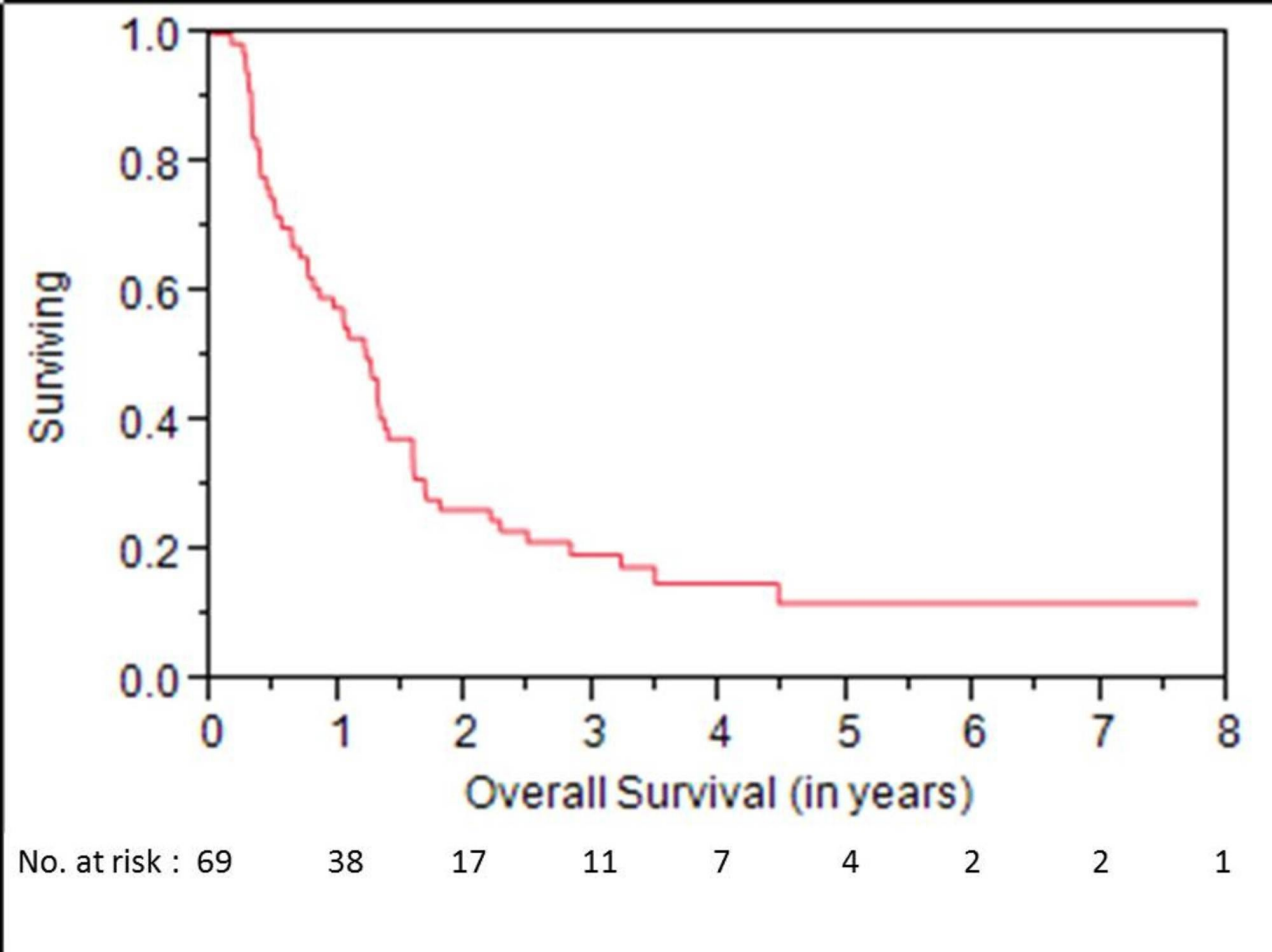
Overall Survival for All Patients

**Figure 2:**
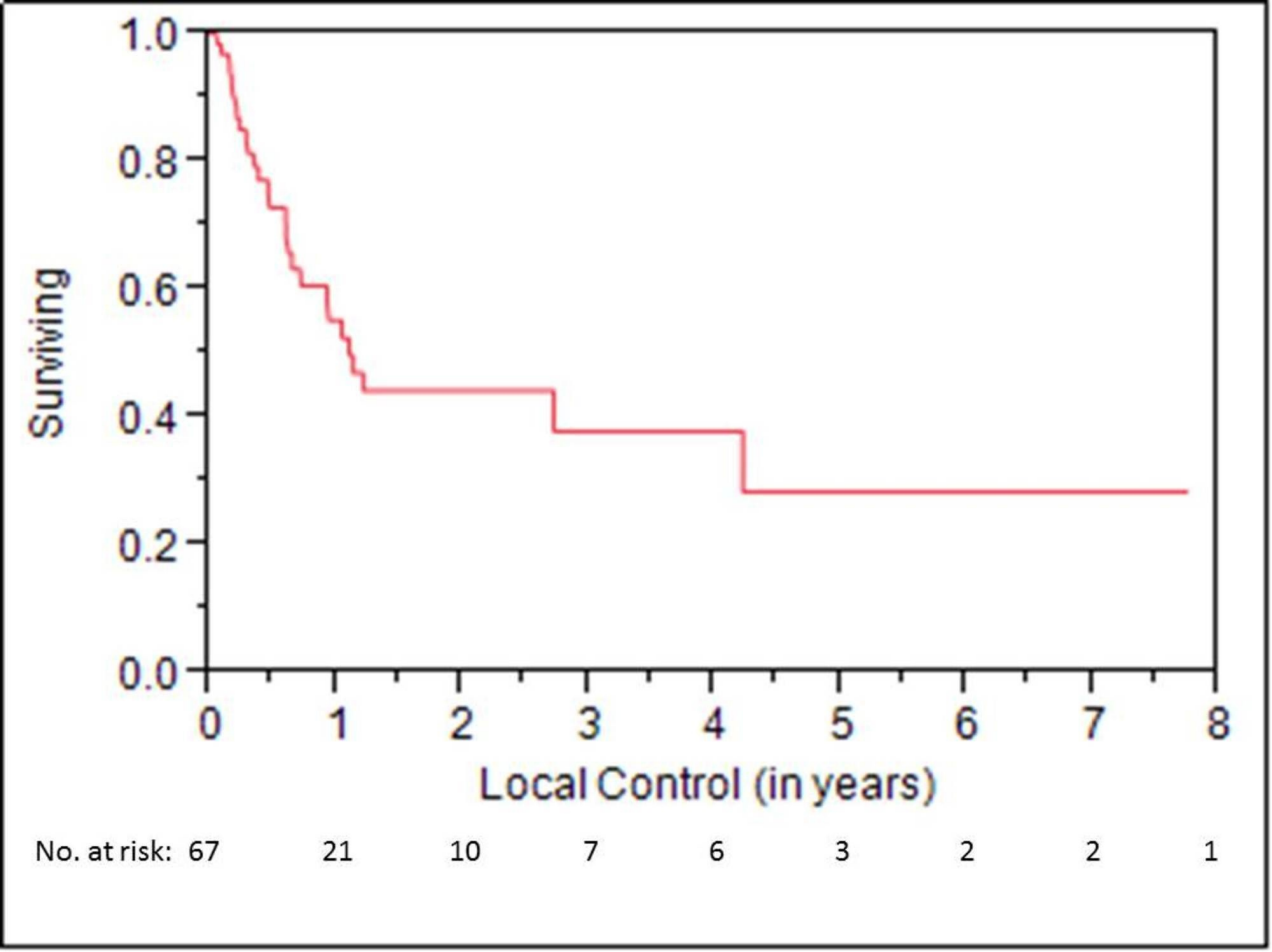
Local Control for All Patients

**Figure 3:**
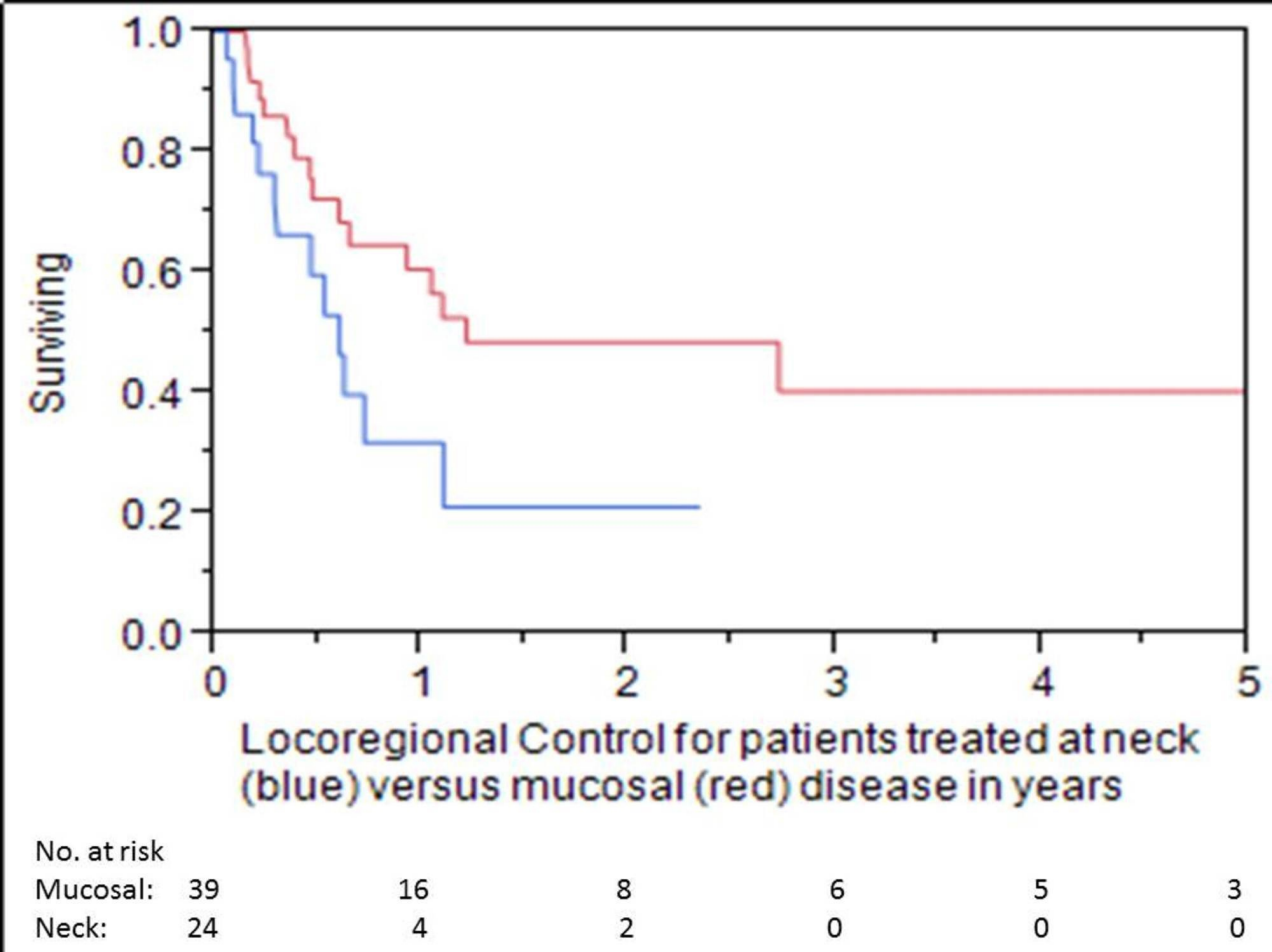
Locoregional Control for Patients Treated at Neck versus Mucosal Disease in Years Blue line = neck disease Red line = mucosal disease

**Figure 4:**
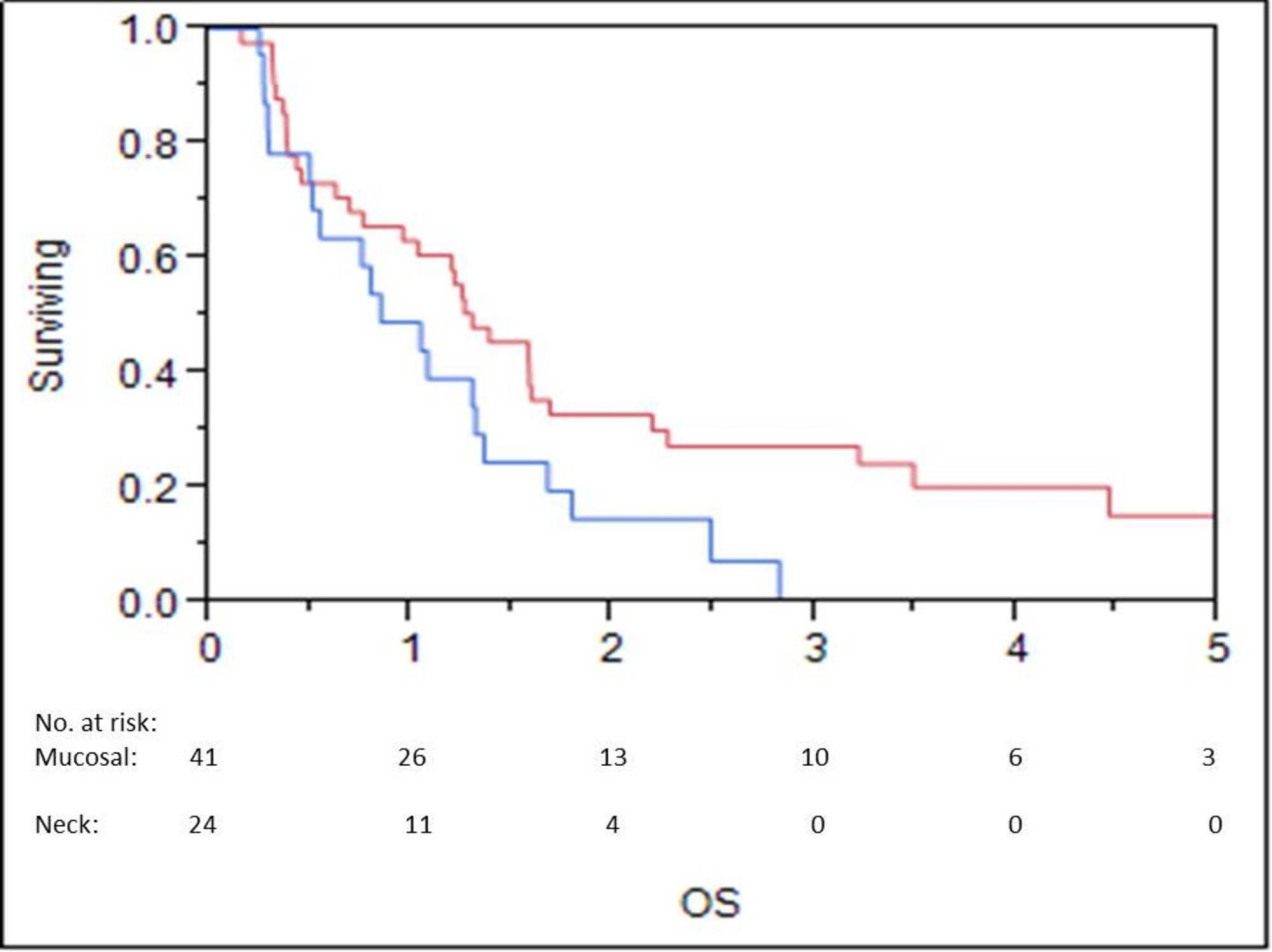
Overall Survival for Mucosal versus Neck Disease Red line = mucosal disease Blue line = neck disease OS: overall survival

**Figure 5:**
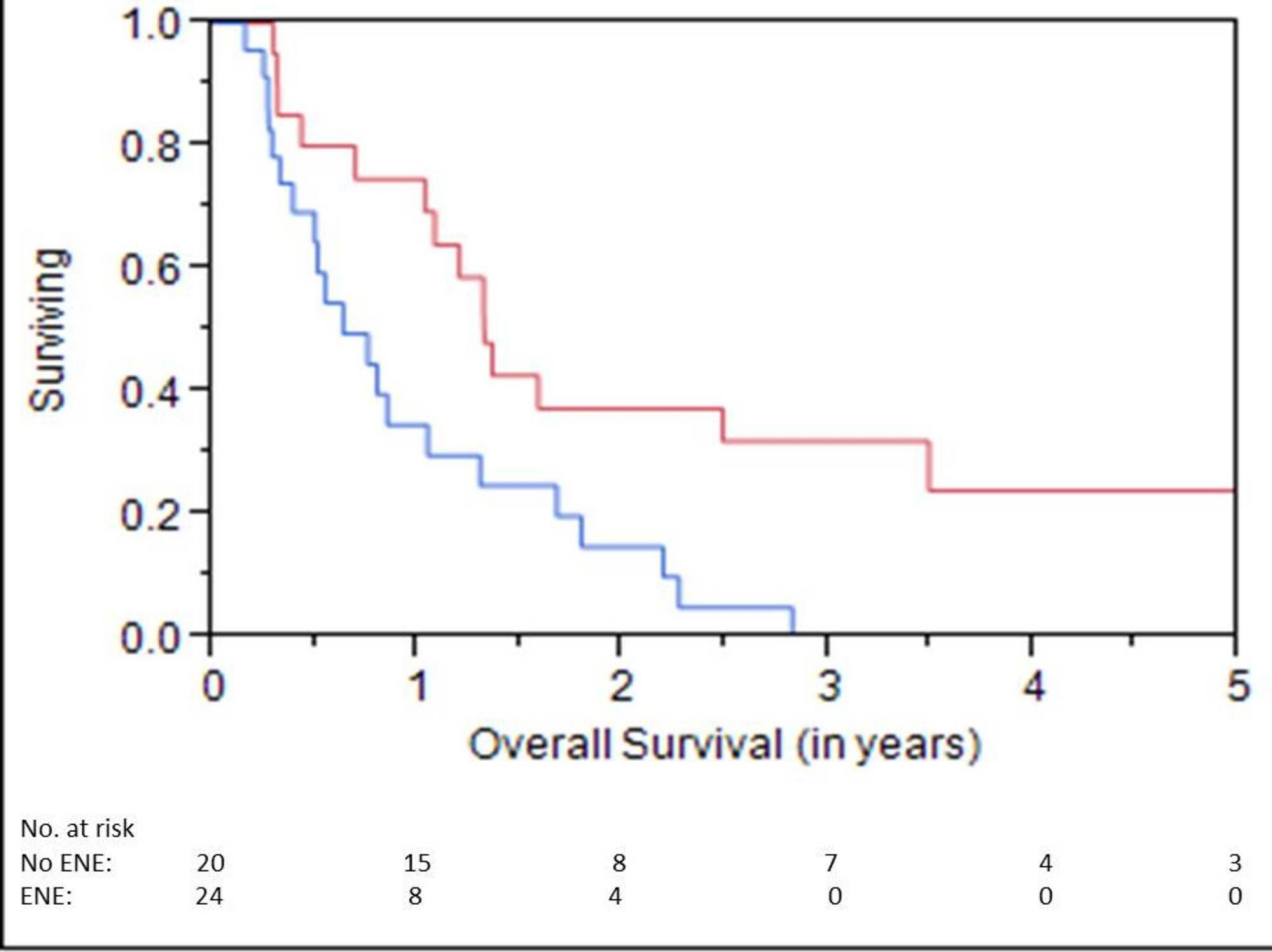
Overall Survival for ENE Blue Line = ENE present Red Line = ENE absent ENE: extranodal extension

OS at one, three, and five years was 58%, 19%, and 12%, respectively (Figure 1). LC at one, three, and five years was 55%, 38%, and 28%, respectively (Figure 2). LRC at one, three, and five years was 50%, 34%, and 25%, respectively. Median time to locoregional failure for patients who received brachytherapy for neck disease and those who received brachytherapy for mucosal sites alone was 0.6 and 1.2 years, respectively (p=.04) (Figure 3). There was a trend towards inferior OS in patients treated for neck disease (median OS 0.8 vs. 1.3 years, respectively), but this did not reach statistical significance (p=.06) (Figure 4). Median OS for patients with ENE at the time of brachytherapy was 0.6 years, compared to 1.3 years for patients without ENE at the time of brachytherapy (p=.007) (Figure 5). Twenty-four of 44 (54%) patients who had lymph nodes dissected at recurrence had ENE. Fifteen of 59 (25%) patients had less than an R0 resection. Patients with an R1 resection had significantly worse OS than those with an R0 resection (RR 2.46, 95% CI 1.22-4.81, p=.01). No patients had an R2 resection.

Seven patients (10%) developed distant metastasis at a median time of 0.46 years after brachytherapy (Table 2).

**Table 2:** Oncologic Outcomes and Toxicities OS: overall survival LC: local control LRC: locoregional control CTCAE: common terminology criteria for adverse events

Median Overall Survival (years)	1.2 (.03-7.7)
1 year OS	58%
3 year OS	19%
5 year OS	12%
Median time to local failure	1.1 (.05-4.1)
1 year LC	55%
3 year LC	38%
5 year LC	28%
Median time to regional failure	0.9 (.05-4.1)
1 year LRC	50%
3 year LRC	34%
5 year LRC	25%
Developed distant metastasis?	n=7 (10%)
Palliation	
Had pain before brachytherapy	n=35
Pain improved after treatment	n=26 (74%)
Acute CTCAE toxicities:	
Any grade 4:	n=0
Any grade 3:	n=18 (27%)
Grade 3 dyspagia or xerostomia	n=18 (27%)
Any grade 2 or 3:	n=48 (72%)
Chronic CTCAE toxicities:	
Any grade 4:	n=2 (3%)
Grade 4 trismus	n=1 (2%)
Grade 4 fibrosis	n=1 (2%)
Any grade 3:	n=12 (19%)
Grade 3 dysphagia	n=7 (11%)
Grade 3 fibrosis	n=2 (3%)
Grade 3 osteonecrosis	n=2 (3%)
Any grade 3-4:	n=14 (22%)
Carotid rupture:	n=5 (7%)

Forty-seven (68%) of patients received brachytherapy for the first recurrence, while the remaining 22 (32%) received brachytherapy for the second or greater recurrence (Table 1). There was no difference in OS between patients being treated for the first recurrence versus patients treated for the second or greater recurrence (RR 1.04, 95% CI .60-1.88, p=.89). A time to first recurrence of less than one year was not associated with significantly worse OS (RR 0.82, 95% CI .44-1.47, p=.52) or local control (RR 1.01, 95% CI .42-2.25, p=0.98). A disease-free interval of greater than one year before brachytherapy was associated with significantly improved local control (63% vs. 39% at one year, p=.0437), but not OS (69% vs. 31% at one year, p=0.25)

Palliation

Of the 35 patients with uncontrolled pain before treatment, 26 (74%) had improvement in their pain following treatment. Of the 26 who had improvement in pain, 21 received intraoperative brachytherapy (IOBT) at the time of resection, while the remaining five had successful palliation after receiving brachytherapy alone.

Adverse events

Adverse events are listed in Table 2. A carotid rupture occurred in five patients. Four of these patients had mucosal sites treated with brachytherapy, and two were treated at neck sites (one patient treated at both mucosal and neck sites). All five patients who had carotid rupture had neck dissection at the time of brachytherapy. For three of these patients, stripping of the carotid artery was specifically mentioned in the operative note. Furthermore, in these cases, the brachytherapy mesh was applied directly over the carotid artery as part of the resection bed. Median time from brachytherapy to rupture was 118 days. Two patients had the rupture in the first two weeks after surgery. After the carotid rupture, median survival was 92 days. One patient died the day of the rupture, and another died three days later. One patient with rupture is still alive at long-term follow-up.

## Discussion

In this modern cohort of patients, we were able to demonstrate favorable outcomes associated with brachytherapy-based re-irradiation of head and neck cancers. Several notable findings emerged from this study, which may help determine which patients benefit most from this approach.

The one-year OS of 58% is consistent with that seen in the literature, although variability in patient selection makes comparisons difficult [12-13]. More importantly, the three-year OS of 19% again suggests that a minority of patients will be long-term survivors, indicating a utility to this approach. However, this survival is inferior compared to the 20% five-year survival reported in patients treated with EBRT re-irradiation in a recent study from Bots et al. [14]. Patient selection may have contributed to this difference, as the patients included in our study often were not felt to be candidates for further EBRT. The one-year LRC rate of 50% that we report is also generally consistent with what is seen in the literature [12-13]. Importantly, there was a substantial difference in LRC in patients treated for primary mucosal tumors versus those treated for neck disease (1.2 vs 0.6 years, respectively, p=.04), as well as a trend towards worse survival in patients with neck recurrences (OS 1.3 vs. 0.8 years, p=.06). These findings must be considered as only hypothesis generating, as a multivariate analysis was not performed. This area needs further study to examine whether the risk of microscopic infiltration in the neck is more significant than in the primary tumor sites, which could mean that irradiating a larger area of the neck with brachytherapy may prove more beneficial. Additionally, patients with ENE in the neck did particularly poorly, with no long-term survivors; brachytherapy in these patients was essentially local palliation.

Our data also support the importance of LC in this poor prognosis group of patients. Only 10% of patients developed distant metastases, reaffirming the importance of local disease control in the head and neck. This is less than the 27% rate of distant failure in this set of patients reported by Salama et al. [15]. Additionally, our data also suggest a palliative benefit in terms of pain. Of the patients who were noted to have pain before treatment, nearly 75% saw an improvement in pain with therapy, which was predominantly resection with brachytherapy.

Our results also confirm the findings of other studies, namely, that patients treated with less than a one-year interval between courses of irradiation had a trend towards worse control and survival outcomes [7,16]. A report by Teckie et al., which also looked at patients receiving IOBT for recurrent head and neck cancers, similarly showed worse LC with a disease-free interval shorter than one year [17].

Our results did show the expected toxicity profile with 22% of the patients experiencing grade three or four late toxicity [13]. Notably, there was a substantial risk of carotid rupture (also called carotid blowout) with five ruptures (7%) among the group, higher than the 2.6% rate previously reported by McDonald et al. [18]. This relatively high rate can likely be attributed to the associated surgical stripping of the carotid artery before the application of brachytherapy, sometimes directly to the adventitia of the vessel. In three of these cases, the disease had to be stripped from the carotid, which likely contributed to the risk as well, especially in light of the fact that two of the carotid ruptures occurred within two weeks of surgery, at which time, only a modest amount of radiation had been delivered. Still, these results highlight the risks of this approach. More recently, our group’s approach has changed to place a flap of tissue above the carotid vessels, in between the brachytherapy seeds, in the hope that this may decrease the risk of injury.

The largest series utilizing low-dose-rate (LDR) brachytherapy to date was performed by Puthawala et al., who reviewed the records of 220 patients treated between 1979 and 1997 for the recurrence of previously irradiated head and neck cancers [13]. At a minimum six month follow-up, local tumor control was achieved in 77% (217/282) of the implanted tumor sites. The two- and five-year disease-specific survival rates for the entire group were 60% and 33%, respectively. The OS for the entire group at five years was 21.7%. Moderate to severe late complications occurred in 27% of the patients. Our study had no minimum follow-up, in order to assess for peri-operative mortality and early failure, and did not have a maximum disease size, which likely led to the difference in OS.

More recently, Grimard et al. published results of 45 patients treated with LDR brachytherapy as the primary or adjuvant treatment for the first recurrence of head and neck cancer [12]. LC at one and two years was 50% and 37%, respectively. Median survival after brachytherapy was 16 months.

The strengths of the current study include its modern patient cohort, the inclusion of patients without a minimum follow-up, and the assessment of palliative outcomes. There are a number of weaknesses of this small, retrospective study that must be acknowledged, including patient selection bias, limitations assessing toxicities, uneven follow-up, and changing treatment paradigms. This data is from one academic center, and the results may not apply to other settings. HPV status was unknown for the majority of patients. The tumors and treatments used were heterogeneous. Three acinic cell carcinoma and one pleomorphic sarcoma were included. Brachytherapy was applied using the judgment of the radiation oncologist and without pre-operative dosimetry, resulting in varying doses and incomplete treatment information. Dosing information was only available for 55% of patients, making an assessment of the optimal brachytherapy dose unfeasible. Finally, the statistical methods used were less than optimal. Competing risks methodology could not be used because of the limitations of the dataset. Only a univariate analysis was performed, so all results must be considered hypothesis generating rather than definitive.

## Conclusions

Our results suggest that brachytherapy is a viable option for certain cases of head and neck cancers in previously irradiated areas. Specifically, our data indicate that brachytherapy may be better suited for mucosal disease than neck disease, particularly in patients with known ENE who have especially poor outcomes. Future directions for research include prospective data collection on dosing, toxicities, and outcomes associated with brachytherapy for head and neck cancers in previously radiated areas.
